# Early Outcomes of Thoracofemoral Bypass for Aortoiliac Occlusive Disease: A 10-Year Single-Center Experience

**DOI:** 10.1055/s-0045-1809704

**Published:** 2025-06-12

**Authors:** Anil Sharma, Sunil Dixit, Mohit Sharma, Sourabh Mittal, Apurva Shah, Shefali Goyal

**Affiliations:** 1 Department of Cardio-Vascular and Thoracic Surgery, Sawai Man Singh Medical College, Jaipur, Rajasthan, India

**Keywords:** atherosclerosis, peripheral vascular disease, thoracobifemoral bypass

## Abstract

**Introduction:**

Thoracofemoral bypass is primarily utilized as a secondary intervention for juxtarenal aortoiliac occlusive disease, with limited instances of its application as an initial treatment, leading to uncertain long-term outcomes. This analysis aims to scrutinize the 10-year experience and early outcomes of 90 patients who underwent thoracofemoral bypass as a primary procedure.

**Materials and Methods:**

A retrospective analysis was conducted on patients undergoing thoracofemoral bypass for severe aortoiliac occlusive disease between August 2012 and August 2022. The primary indication was complete abdominal aorta obstruction at the renal artery level with an unsuitable site for aorta clamping. The BARD IMPRA expanded polytetrafluoroethylene vascular graft was employed for thoracobifemoral bypass surgery.

**Results:**

Among the 90 patients, 83 (92.22%) were male, and 7 (7.78%) were female, with ages ranging from 51 to 77 years. Intraoperative and postoperative data were analyzed, and the mean follow-up duration was 30 days. The 30-day mortality rate was 3.33% ( *n* = 3). Major morbidities included graft occlusion in one patient, managed by embolectomy, and ascites in another patient, addressed conservatively.

**Conclusion:**

This study demonstrates that thoracic aorta to femoral artery bypass, as a simple extra-anatomic bypass technique, can yield favorable outcomes when chosen as the initial treatment for patients with juxtarenal total aortoiliac occlusive disease. Thoracofemoral bypass exhibits a safe, acceptable outcome with reliable patency.

## Introduction

The management of complete obstruction of the abdominal aorta at the renal artery level poses a formidable surgical challenge. Traditionally, the descending thoracic aorta (DTA) has been employed as an inflow source, primarily in cases of graft failure, infection, or intra-abdominal pathologies precluding a standard abdominal aortic approach. While endovascular techniques have emerged as effective treatments for symptomatic aortoiliac occlusive disease (AIOD), they exhibit lower mortality and morbidity compared with open surgery. However, the primary patency rates of endovascular approaches are inferior to those of surgical grafts. 1

Surgical intervention remains the preferred choice, especially for cases of AIOD originating at the juxtarenal level and extending to the bilateral iliac arteries. Aortofemoral bypass (AFB) may encounter technical challenges and increased postoperative risks due to inadequate anastomotic areas and severe calcification. 2 3 Although extensive endarterectomy can create sufficient anastomotic space, residual calcification and abnormal arterial walls postendarterectomy pose significant challenges to successful anastomosis. Moreover, aortic cross-clamping, both suprarenal and infrarenal, in this pathological region may result in complications affecting visceral organs and the kidneys. Extra-anatomic bypass or thoracofemoral bypass (TFB) serves as a viable alternative to mitigate these complications. 4 5

While the long-term results of extra-anatomic bypass vary across published series, TFB is commonly reserved as a secondary surgical option following failures of open aortic revascularization and aortofemoral graft infections. 4 However, considering the challenges posed by pararenal aortic neck calcification or thrombus hindering the safe construction of the proximal anastomosis of AFB, TFB can also be contemplated as a primary surgical option. 5 6 In our institution, we have adopted TFB as the initial treatment for severe AIOD, and in this study, we aim to present the outcomes of cases treated with TFB and share our surgical experience spanning the past 10 years.

The utilization of the DTA as an alternative inflow source for AIOD was introduced in 1961 by Stevenson et al and Blaisdell et al. 7 8 Initially employed as a remedial reconstruction for aortic graft failure, graft infection, or other intra-abdominal catastrophes, bypass grafting from the DTA to the iliac or femoral arteries has evolved over time. The purpose of this study is to report early results (30 days) encompassing 90 DTA to femoral artery bypass grafting procedures performed at our institution.

## Materials and Methods

**Fig. 1:**
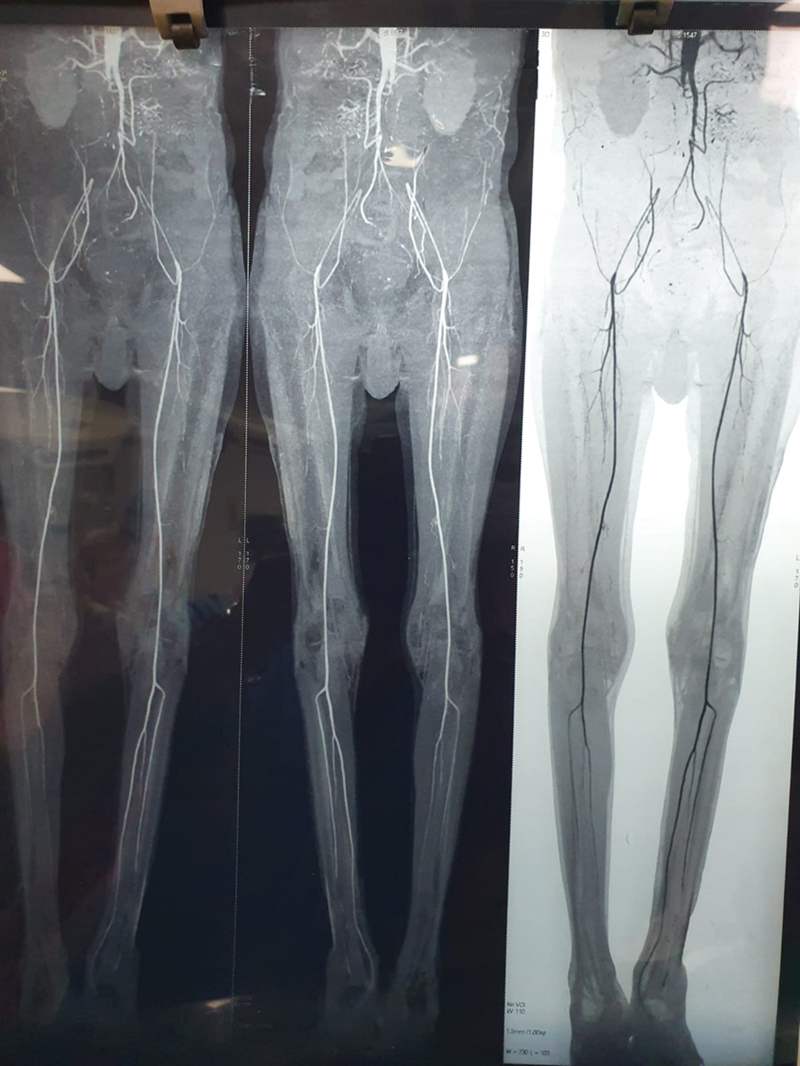
Preoperative computed tomography image.

A total of 90 patients with AIOD underwent descending thoracic aortobifemoral bypass as primary procedure at S.M.S. Medical College and Group of Hospitals, Jaipur (Rajasthan) between August 2012 and August 2022. Demographic information and procedural details were extracted from hospital records. Angiography, available for all patients, was performed and subsequently reviewed ( Fig. 1 ).

Medical conditions, indications for surgery, and any prior vascular interventions were comprehensively assessed through hospital records. Prior to the operation, complete angiography of the aortoiliac and lower extremity vessels was conducted using standard techniques. Additionally, coronary angiography was systematically performed in all patients to exclude the presence of coronary artery disease.

For patients with suspected limited pulmonary reserve or documented severe pulmonary disease—a potential contraindication to the procedure—pulmonary function tests and baseline arterial blood gas measurements were obtained. DTA to femoral artery bypass grafting served as the primary procedure for patients without previous direct aortoiliac or extra-anatomic reconstructions.

The criteria for selecting patients for primary repair included (1) severe atherosclerotic disease or complete occlusion of the infrarenal aorta with relative contraindications to direct aortic reconstruction (e.g., prior intra-abdominal sepsis, multiple abdominal operations, radiation, or colostomy) and (2) severe atherosclerotic disease or complete occlusion of the infrarenal aorta, wherein the DTA was deemed the preferred source of inflow by the operating surgeon based on the severity of occlusive disease in the infrarenal aortic segment.

Surgery was indicated in all patients presented with vascular symptoms like disabling intermittent claudication, chronic nonhealing ulcers, and rest pain. Computed tomography (CT) angiography revealed infrarenal aortic stenosis, juxtarenal aortic occlusion, and stenosis of the common and external iliac arteries in all cases ( Fig. 1 ).

**Fig. 2:**
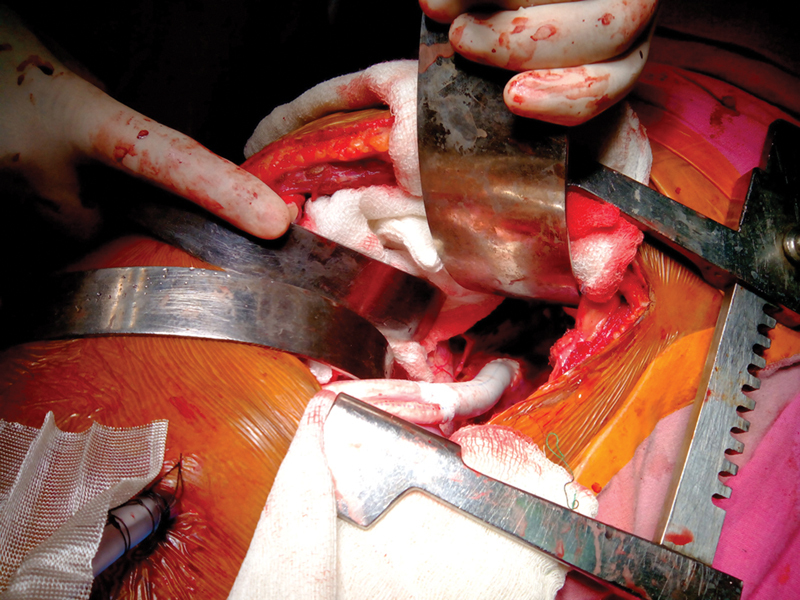
Intraoperative image.

Considering the absence of a suitable site for aortic cross-clamping, abdominal aortobifemoral bypass was deemed hazardous. The BARD IMPRA expanded polytetrafluoroethylene vascular graft, along with the Vascutech graft, were employed for bypass surgery ( Fig. 2 ).

### Surgical Technique

Patients underwent general anesthesia with continuous hemodynamic monitoring during the surgical procedure. Although not mandatory, a double-lumen endotracheal tube was considered advantageous. The patient was positioned with the left hemithorax elevated at an angle of 30 to 45 degrees, and the pelvis was maintained as flat as possible to facilitate access to both groins. Adequate preparation and draping were performed on the chest, abdomen, and both groins.

An anterolateral thoracotomy was conducted through the 7th intercostal space. The proximal anastomosis of a bifurcated graft was executed in an end-to-side fashion at the lower DTA, as illustrated in Fig. 2 . Subsequently, the graft limbs were guided through a tunnel between the rectus abdominis muscle and peritoneum to a short midline incision at the level of the umbilicus. Each limb of the graft was then drawn through a subcutaneous tunnel to reach the respective sides of the groin, where anastomosis to each common femoral artery was performed.

**Fig. 3:**
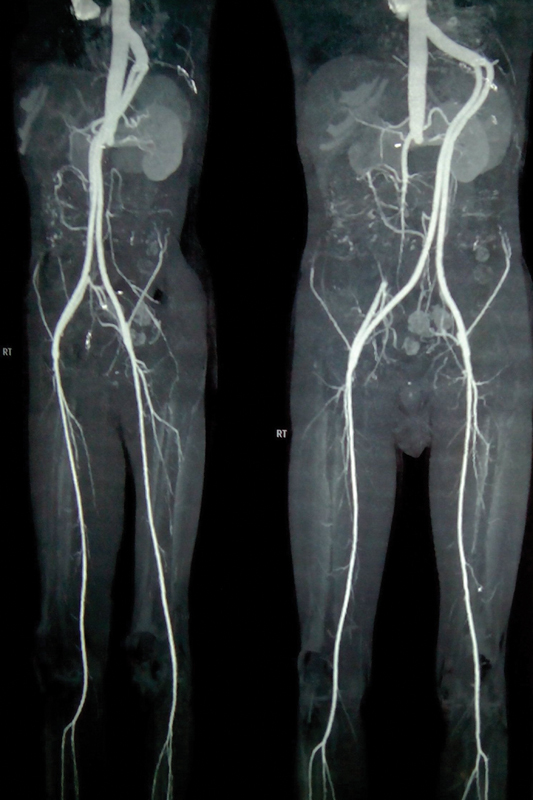
Postoperative image.

In the postoperative period, thorough assessments were conducted to evaluate distal pulsations, primary patency, warmth of the foot, symptom relief, wound infection, healing of ulcers, and the occurrence of complications. Additionally patients were counseled for smoking cessation and vascular protective measures. Follow-up CT angiography was systematically performed to assess the vascular graft's performance. Fig. 3 depicts a CT angiography revealing a normal flow pattern in the graft.

## Results

Ninety patients underwent this surgery in S.M.S. Hospital, Jaipur during study period. Out of 90 patients, 83 (92.22%) were males and 7 (7.78%) were female. The age range spans from 51 to 77 years, with an average age of 58.62 ± 6.647 years. Among 90 study participants, 82 (91.11%) had coronary artery disease (CAD), whereas 90 (100.00%) suffered from hypertension. Diabetes mellitus (DM) affected 54 patients (60.00%), and hyperlipidemia was prevalent in 86 patients (95.56%). Additionally, 14 patients (15.56%) had renal disease, 7 (7.78%) had undergone prior intra-abdominal surgery, and 1 (1.11%) had a history of prior thoracic surgery. Pulmonary disease was observed in 38 patients (42.22%) ( Table 1 ).

**Table 1:** Baseline variables of study participants

Variable	*n*	%
Sex		
Male	83	92.22
Female	7	7.78
Age		
Range (y)	51–77	
Mean ± SD (y)	58.62 ± 6.647	
Associated morbidities		
CAD	82	91.11
Hypertension	90	100.00
DM	54	60.00
Hyperlipidemia	86	95.56
Renal disease	14	15.56
Prior intra-abdominal surgery	7	7.78
Prior thoracic surgery	1	1.11
Pulmonary disease	38	42.22

Abbreviations: CAD, coronary artery disease; DM, diabetes mellitus.

Average duration of surgery falls within the range of 2.5 to 4.5 hours, with a mean duration of 3.33 hours and a standard deviation of 0.537. Blood loss during surgery was 100 to 500 mL, with an average of 290 mL and a standard deviation of 97.65.

Postoperative findings revealed that the extubation time after surgery spans from 6 to 12 hours, with an average time of 8.74 hours and a standard deviation of 1.608. Intensive care unit stays ranged from 3 to 5 days, with an average stay of 3.64 days and a standard deviation of 0.724. Reexploration after surgery was required in 3 (3.33%) cases. Rare postoperative complications included renal failure, myocardial infarction, peritonitis, fever, wound complications, and mortality, each occurring in 1.11% of the cases ( Table 2 ).

**Table 2:** Intra- and postoperative findings of study participants

Outcome	Range/number	Mean ± SD/%
Intraoperative findings		
Avg duration of surgery	2.5–4.5 h	3.33 ± 0.537
Blood loss	100–500 mL	290 ± 97.65
Graft used		
PTFA	49	54.44
Vascutech	41	45.56
Postoperative findings		
Postoperative extubation time	6–12 h	8.74 ± 1.608
ICU stay	3–5 d	3.64 ± 0.724
Reexploration	3	3.33
Renal failure	1	1.11
MI	1	1.11
Peritonitis	1	1.11
Fever	6	6.67
Wound complications	5	5.56
Mortality	3	3.33

Abbreviations: Avg, average; ICU, intensive care unit; MI, myocardial infarction; PTFA, polytetrafluoroethylene; SD, standard deviation.

Among the 90 patients we performed concomitant coronary artery bypass grafting (CABG) (left internal mammary artery to left anterior descending artery) via thoracotomy in two patients during the same procedure.

Thirty-day follow-up of 87 individuals remaining after early mortality of three patients. Data revealed a 100% graft patency rate. All grafts remained open and functional during this period. There were only three immediate postoperative mortalities. These positive findings in graft patency and the absence of subsequent mortality underscore the favorable short-term outcomes and effectiveness of the surgical interventions performed on the individuals in this cohort ( Table 3 ).

**Table 3:** Findings of 30-day follow-up of surviving study participants

30-day follow-up	*n* (out of 87)	%
Graft patency	87	100
Mortality	0	0

## Discussion

Several notable limitations characterize this study, foremost among them being its retrospective design and the absence of randomization, primarily due to the infrequent occurrence of the condition under investigation. Consequently, drawing unequivocal conclusions from a single-center experience is challenging. Nevertheless, this study stands out among the limited existing literature by presenting a substantial number of cases where the TFB procedure was chosen as the primary treatment for specific patients. A major limitation of our study is the 30-day endpoint follow-up, whereas these patients required prolonged follow-up for optimal results. The study's significance is further underscored by its comprehensive follow-up results and the wealth of surgical expertise accumulated over approximately 10 years.

In the current landscape where endovascular approaches are increasingly favored for AIODs, the durability of open surgical reconstruction remains a pivotal consideration. Our study identifies specific scenarios warranting careful consideration for the utilization of open TFB, including severe circumferential juxtarenal/suprarenal aortic calcification, failed AFB, or an initial endovascular approach lacking favorable anatomy for direct reconstruction. This paper underscores the procedure's low perioperative morbidity, mortality, and favorable patency rates, supporting its application not only as an alternative but also as a primary revascularization option in select cases. 9

The presented study encompasses a 10-year experience involving 90 TFB procedures conducted at a single institution, representing a substantial accumulation of clinical data. Traditionally reserved for cases of aortic graft failure or infection or as an alternative when a direct transabdominal aortic approach is unfeasible, bypass grafting from the descending thoracic to femoral arteries has yielded satisfactory results in prior publications, with patency rates ranging from 76 to 86% at 5 years. 10

While DTA-to-iliofemoral artery bypass grafting is firmly established as a secondary procedure, its role as a primary operation remains contentious. In carefully selected patients with challenging abdominal conditions, where the infrarenal aorta's approach is complicated, primary DTA-to-iliofemoral artery bypass grafting emerges as a preferable alternative. 11

It is imperative to contextualize historical series, considering that patients undergoing revascularization for AIOD via direct AFB grafting several decades ago often presented with less severe atherosclerotic disease and a lower prevalence of limb-threatening ischemia.

The utilization of the DTA as an inflow source for primary aortoiliac reconstruction offers several advantages over conventional direct aortic repair. Notably, the DTA typically exhibits minimal atherosclerotic disease, rendering it more suitable for proximal anastomosis than the abdominal aorta.

While AFB grafting remains the standard surgical treatment for AIOD, alternative procedures such as axillofemoral bypass or thoracobifemoral bypass are considered when abdominal aortic surgery is contraindicated due to severe inflow site disease. In this context, thoracobifemoral bypass holds major advantages over axillofemoral bypass, offering superior inflow, requiring a shorter graft length, providing enhanced graft protection from infection and mechanical trauma, and demonstrating a superior patency rate.

Our series presented unique considerations, such as the concurrent recommendation of CABG followed by TFB in patients with coronary artery disease. Simultaneous CABG via thoracotomy approach and TFB were performed in select cases, with one postoperative mortality due to myocardial infarction. 12 13

In contrast to other series, our study adopted a distinct graft tunneling approach, directing the graft anteriorly through the anterior aspect of the diaphragm to a short midline incision, then to both femoral arteries. All patients in our series demonstrated good distal pulses, and the operative mortality rate was notably low at 3.33%, with one mortality attributed to myocardial infarction.

Graft failure or occlusion, reported in 4 to 30% of cases over 3 to 5 years in other series, was managed effectively in our series through embolectomy, resulting in good distal flow and favorable patient recovery. Overall, our series demonstrated superior inflow and more reliable patency with thoracobifemoral bypass, recommending its consideration in selected patients where conventional approaches to the abdominal aorta are deemed hazardous ( Tables 1 and 2 ).

## Conclusion

In individuals presenting with total aortic lesions at the juxtarenal level, both AFB and endovascular interventions may pose technical challenges and carry substantial morbidity risks, primarily attributed to the nature and location of the occlusive lesions. In light of these considerations, TFB emerges as a comprehensive primary revascularization approach for patients with this specific lesion profile. Our study provides evidence supporting the efficacy of TFB as an initial treatment option in carefully selected patients with juxtarenal total aortic occlusion.

Despite the inherent complexity of the surgical technique involved in TFB, our findings reveal that it can deliver favorable outcomes when chosen as the primary treatment in this subset of patients. Notably, TFB exhibits a favorable safety profile, with acceptable rates of mortality and morbidity.
